# Challenges Associated with Tyrosine Kinase Inhibitor Therapy for Metastatic Thyroid Cancer

**DOI:** 10.4061/2011/985780

**Published:** 2011-10-04

**Authors:** Maria E. Cabanillas, Mimi I. Hu, Jean-Bernard Durand, Naifa L. Busaidy

**Affiliations:** ^1^Department of Endocrine Neoplasia and Hormonal Disorders, The University of Texas MD Anderson Cancer Center, Houston, TX 77030, USA; ^2^Department of Cardiology, The University of Texas MD Anderson Cancer Center, Houston, TX 77030, USA

## Abstract

Tyrosine kinase inhibitors (TKIs) which target angiogenesis are promising treatments for patients with metastatic medullary and differentiated thyroid cancers. Sorafenib, sunitinib, and pazopanib are commercially available drugs which have been studied in these diseases. Vandetanib is the first drug approved in the United States for treatment of medullary thyroid cancer. These TKIs are used as chronic therapies, and therefore it is imperative to understand the adverse event profile in order to avoid excessive toxicity and maintain patients on therapy as long as it proves beneficial. Here we review common toxicities, management of these, and other challenging situations that arise when using TKIs in patients with thyroid cancer.

## 1. Introduction

Thyroid cancer is now the 5th most commonly diagnosed cancer in women and 9th in overall incidence in the United States; however, fewer than 2000 people die per year of their disease and mortality rates have remained fairly stable for the past several decades [1]. The most common form of thyroid cancer, differentiated thyroid cancer (DTC), is derived from the follicular cells of the thyroid, and it includes papillary and follicular thyroid cancers. While most patients are cured or have indolent disease, a small percentage develop metastases that no longer respond to treatment with radioactive iodine or TSH suppressive therapy. Medullary thyroid cancer (MTC) accounts for only about 2-3% of thyroid cancers and is derived from the neuroendocrine “C” cells of the thyroid gland. The only treatment with curative intent for medullary thyroid carcinoma is complete surgical resection.

Therapy with tyrosine kinase inhibitors (TKIs) has only recently been studied in thyroid cancer. The discovery that BRAF (in papillary and anaplastic thyroid cancers) and RET (in MTC) mutations, as well as angiogenesis, play a significant role in tumorigenesis in DTC and MTC led to several clinical trials over the past decade with multikinase inhibitors. For purposes of this paper, TKIs refer to small molecule drugs, which target multiple pathways, including, but are not limited to, vascular endothelial growth factor receptor (VEGFR). Sorafenib, sunitinib, and pazopanib are three commercially available TKIs which have shown favorable results in phase II trials in DTC [2–4]. Although these small trials have reported favorable responses, at this time, there are no published results of large phase III trials in DTC. Favorable results of a phase III, randomization study of vandetanib versus placebo in MTC have been reported [5]; however, it is important to note that patients on this study were not required to have progressive disease prior to study entry. Vandetanib was recently approved by the Food and Drug Administration for symptomatic or progressive MTC, establishing it as the first drug to be approved for this disease. The drug is available only through the Vandetanib Risk Evaluation and Mitigation Strategy (REMS) Program due to the prolongation of the QT interval and reported cases of torsades de pointes and sudden death in clinical trials. Sorafenib has also been studied in MTC in a phase II trial [6], and encouraging results of sunitinib in MTC have been presented at a national meeting [7]. 

There are many challenges posed by the use of TKIs, which we believe should be used with caution and reserved for patients with either advanced, progressive disease or bulky disease which may compromise organ function. This review focuses on highlighting the most common and problematic adverse events associated with TKIs with suggestions for management. Other dilemmas that often arise with use of these drugs will be described as well.

## 2. Adverse Event Management

Although TKIs are generally better tolerated than cytotoxic chemotherapy, many patients develop side effects from on-target and off-target effects which require aggressive management in order to maintain patient compliance, optimize therapy, and avoid potentially life-threatening consequences. Since many patients require long-term use of TKIs for continued control of disease, it is imperative for the treating clinician to be familiar with the potential side effects of these drugs. The most frequent side effects of TKIs are hypertension, dermatologic effects, fatigue, and diarrhea. In addition, the risk of bleeding and liver toxicity may be fatal. The clinician should conduct thorough physical and laboratory examinations prior to considering therapy with these drugs to identify the most appropriate choice of treatment and must monitor and treat adverse events during therapy. Treatment of all comorbid conditions should be optimized and drug-drug interaction, antifungals, antiemetics, and class III antiarrhythmic agents avoided to prevent interactions with TKIs. In this section we will discuss the most common and potentially fatal side effects of TKIs with management recommendations. 

Table 1 lists adverse events of the commercially available TKIs relevant to thyroid cancer, their incidence, and grades (data extracted from phase III trials in renal cell carcinoma and package inserts) using Common Terminology Criteria for Adverse Events version 3.0 (CTCAE v3.0). The CTCAE is a list of descriptive terminology utilized for adverse event grading and reporting on clinical trials and is made available through the CTEP website at http://ctep.cancer.gov/protocoldevelopment/electronic_applications/docs/ctcaev3.pdf. 

### 2.1. Drug-Drug Interactions

Cytochrome P450 enzymes, expressed primarily in the liver, play a primary role in the metabolism of many drugs. Sunitinib, sorafenib, pazopanib, and vandetanib are all metabolized by cytochrome P450 3A4 (CYP3A4). Of the four drugs, sorafenib appears to be the least susceptible to CYP3A4 inducers or inhibitors, although the package labeling warns against concomitant use of CYP3A4 inducers [8]. Concomitant use of CYP3A4 inducers may decrease the plasma concentration of the TKI, resulting in decreased efficacy, while inhibitors may increase the plasma concentration, resulting in toxicity. Itraconazole, a potent inhibitor of CYP3A4, does not appear to affect the metabolism of vandetanib [9].  Table 2 lists the more common, clinically significant drugs metabolized via the CYP3A4 enzyme system.

The medical history should include a thorough review of medications which may affect the metabolism of the TKI. Concomitant drugs which are metabolized via CYP3A4 should be avoided or substituted for another drug. If a CYP3A4 inhibitor drug cannot be eliminated, a dose reduction in the TKI should be considered. Patients should also be monitored for increasing side effects if a CYP3A4 inhibitor is coadministered.

### 2.2. Cardiovascular

Hypertension is the most common cardiovascular side effect associated with antiangiogenic drugs. The mechanism of hypertension is not well understood, but it has been suggested that it is due to increased fluid retention, endothelial dysfunction, nitrous oxide inhibition, rarefaction [10], reduction of vascular surface area, and increase in peripheral vascular resistance caused by inhibition of angiogenesis [11–14]. A recent study by Rini et al. suggests that the rise in blood pressure above 140/90 may be a biomarker for anticancer therapy and was associated with significant survival benefit even with treatment of antihypertensives. The use of antihypertensives did not reduce the efficacy of sunitinib in metastatic renal cell carcinoma [15].

The onset of hypertension is variable. Blood pressure may begin to rise within days of therapy prior to steady state or the onset of the therapies' biological effects or may be more indolent. There are no clear guidelines for managing TKI-induced hypertension. It is our clinical practice to use ACE inhibitors, angiotensin receptor blockers (ARBs) or a beta blocker as first-line therapy for hypertension since these drugs are not metabolized via the CYP3A4 enzyme system. However, the choice of an antihypertensive should be individualized. The Angiogenesis Task Force of the National Cancer Institute Investigational Drug Steering Committee recently published guidelines for management of hypertension with TKIs [16]. Hypertension should be controlled based on compelling and noncompelling indications to a goal of <140/90 prior to starting TKIs. Once a TKI is initiated, patients should have the blood pressure monitored within 1 week. Blood pressure monitoring at home may be more effective at prediction of outcomes from cardiovascular disease than clinic blood pressure monitoring [17]. If the blood pressure is above goal, antihypertensive therapy should be initiated or adjusted. Patients should continue to check their blood pressure daily (with brachial blood pressure device) and report results on a weekly basis (until adequate blood pressure control is achieved), and antihypertensive drugs should be rapidly titrated or new drugs added to the regimen. Once control of blood pressure is obtained, the blood pressure should be monitored on a monthly basis. Interruption or dose reduction of the TKI may be necessary in order to achieve adequate blood pressure control. Some calcium-channel blockers, such as felodipine, diltiazem, nifedipine, and verapamil, are CYP3A4 substrates or inhibitors and should be avoided.

Sunitinib and pazopanib can lead to QT interval prolongation; therefore, they should be used with caution in patients with a history of QT prolongation and patients taking antiarrhythmic drugs. Torsade de pointes was seen in <0.1% of patients exposed to sunitinib and <2% of patients treated with pazopanib. Vandetanib carries a black box warning due to QT interval prolongation, Torsade de pointes, and sudden death observed in clinical trials involving patients with a broad variety of solid malignancies. Serial monitoring of electrocardiograms and electrolytes is mandated and electrolyte abnormalities should be corrected [9, 18, 19]. In a phase III trial that examined the efficacy and safety of vandetanib 300 mg in the treatment of unresectable locally advanced or metastatic MTC, QT prolongation was reported in 14% of patients randomized to vandetanib and in 1% of patients randomized to placebo, with 8% (18/231) and 1% (1/99), respectively, being ≥grade 3 events. Vandetanib should not be given to patients who have a history of Torsades de pointes, congenital long QT syndrome, bradyarrhythmias, or uncompensated heart failure. Vandetanib should not be started in patients whose corrected QT interval (QTcF, Fridericia formula) is greater than 450 ms. Specific guidelines for monitoring of QT abnormalities and electrolytes in patients taking vandetanib are specified in the package insert [9]. In addition, use of concomitant drugs known to prolong the QT interval, such as amiodarone and erythromycin, should be avoided. 

A less common but serious adverse event associated with TKIs is systolic and diastolic congestive heart failure. It appears to be more common with sunitinib but has been reported with sorafenib and pazopanib. Patients may present with very dramatic symptoms of heart failure, while others demonstrate mild symptoms which may be difficult to differentiate from fatigue due to the TKI or the tumor itself [20]. Cardiac toxicity, although not always completely reversible, is often a manageable condition if patients have careful monitoring and treatment with routine heart failure therapies with beta blockers and ACE inhibitors/ARB as recommended by the guidelines of heart failure management by the American College of Cardiology. The etiology of the heart failure is thought to be due to direct reversible cardiomyocyte toxicity, possibly exacerbated by hypertension which may progress to irreversible, progressive injury if not treated with standard heart failure therapy [21]. This toxicity is not completely understood, but platelet-derived growth factor receptor-*β* (PDGFR-*β*) inhibition has been implicated as playing a role in the response to pressure-overload-induced stress [22]. We recommend that all patients initiating TKIs have a baseline echocardiogram and periodic monitoring while they are on therapy. Furthermore, aggressive management of hypertension may help reduce cardiomyocyte damage.

**Case Number 1**

**Figure 1:**
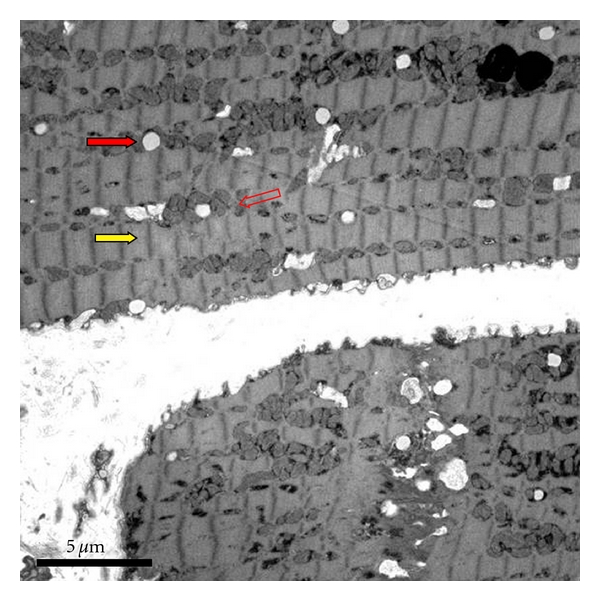
Transmission electron micrographs of endomyocardial biopsy from patient with systolic heart failure treated with a TKI. Section shows hypertrophy and interstitial edema with edematous mitochondria (open red arrow), with increased lipid droplets (solid red arrow) and dilatation of sarcotubular elements (yellow arrow). These findings are consistent with acute but reversible injury.

A 69-year-old woman with a history of hypertension and premature ventricular contractions was referred to our center. She had a history of T4a, N0, M0, stage IVA papillary thyroid cancer for 10 years prior. The patient's thyroid cancer was initially managed with total thyroidectomy and radioactive iodine ablation, but she developed local recurrence and pulmonary metastases several years later. She continued to have progressive disease in the lungs and neck and was referred to our center. The patient was enrolled into a phase II clinical trial with an investigational TKI targeting VEGFRs, PDGFR, and others. The patient's blood pressure was normal prior to initiation of the investigational TKI, but one week later she developed grade 2 hypertension which was difficult to control despite treatment with multiple antihypertensive agents. Her pretreatment echocardiogram demonstrated an ejection fraction of 55–60%. Nearly 4 months after starting on the investigational agent, she underwent adenosine stress test which identified a 30% ejection fraction with hypokinesia in the anterior septal segments which partially reversed with rest. Because of the presence of a left bundle branch block at baseline, definitive diagnosis of ischemia was not possible from the images. Carvedilol was initiated, and the investigational TKI was held. Echocardiogram confirmed the low ejection fraction. A cardiac catheterization with myocardial biopsy was performed. She was found to have mild ischemic heart disease (defined as less than 50% stenosis in any coronary) which was disproportionate to her degree of heart failure, and therefore the heart failure was attributed to the TKI. Direct cardiomyocyte toxicity was confirmed with the biopsy, demonstrating hypertrophy and interstitial edema, increased lipid droplets, and dilatation of sarcotubular elements (Figure 1). Since the biopsy showed no myocyte death (indicating reversibility) and the echocardiogram showed a return to baseline, after 3 weeks, the investigational agent was reintroduced at a reduced dose. Two months later she was found to have progression of disease, and the investigational agent was discontinued permanently.

### 2.3. Renal

Proteinuria associated with antiangiogenic therapies was first described with bevacizumab, a monoclonal antibody against VEGF [23]. Small-molecule tyrosine kinase inhibitors, which inhibit VEGF-R, lead to proteinuria as well [24]. Thrombotic microangiopathy and acute interstitial nephritis have been reported with sorafenib and sunitinib [25, 26]. The glomerular podocytes express VEGF, and glomerular endothelial cells express VEGF receptors. Thus, a proposed mechanism of proteinuria is that deletion of VEGF allele in podocytes or inhibited VEGF signaling leads to proteinuria and capillary endotheliosis [27].

All patients who will receive antiangiogenic therapies should have a baseline urinalysis and protein to creatinine ratio, with routine monitoring for development of proteinuria while on treatment. A urine protein to creatinine ratio of ≥1 or 24-hour urine with ≥1 gram/dL/24 hours of protein should prompt intervention. The decision to hold drug should be considered on a case-by-case basis. Treatment with an ACE inhibitor or ARB should be initiated and consultation with nephrology may be warranted. As proteinuria is a class effect of antiangiogenic treatments, changing from one agent to another may not prevent this effect in a patient.

### 2.4. Dermatologic

Dermatologic reactions observed with TKIs include hand-foot syndrome (HFS), skin induration or callous formation, rash, alopecia, hair texture and color changes, and skin discoloration. HFS, the most common and potentially most debilitating dermatologic effect, presents as desquamating lesions in a palmoplantar distribution typically at pressure points or areas of friction or trauma. The lesions can significantly affect a patient's quality of life, thus necessitating drug discontinuation or dose reduction. The pathogenesis of HFS is not entirely clear. Preventive application of hand and foot lubricants should be implemented at time of drug initiation. The package insert for sorafenib gives clear recommendations on dose modifications and holds for skin toxicity (Table 3). It has been the authors' experience with sorafenib that when patients develop grade ≥3 HFS, drug interruption until skin toxicity declines to grade ≤1 with reinitiation at 200 mg daily, and titration by 200 mg every 3–5 days can prevent further escalation of skin toxicity (unpublished data). Stevens-Johnson syndrome, characterized by a prodrome of malaise and fever, followed by rapid development of erythematous or purpuric macules, which can progress to epidermal necrosis or sloughing, has been reported with vandetanib. A patient with these signs and/or symptoms should discontinue drug therapy immediately and seek medical attention, as this could be a life-threatening adverse effect.

Skin induration and callous formation can lead to pain at pressure points and limit mobility. Referral to podiatry can be considered to reduce callous size. Skin evaluation for development of actinic keratoses or keratoacanthoma-type squamous cell carcinomas (KA-SCC) should be performed regularly while being treated with sorafenib and BRAF inhibitors, as these lesions have been described primarily with targeted therapy against Raf kinase or mutant BRAF [32–35]. These lesions can develop as solitary or multiple lesions, weeks to months after starting drug therapy, and do not need to be confined to sun-exposed areas. Fortunately, KA-SCC has not been reported to metastasize, and spontaneous regression has been reported [32]. KA-SCCs should be completely excised. It has not been uniformly recommended that drug discontinuation occur when KA-SCCs develop due to the low metastatic potential; however, patients should be made aware of this effect and maintain routine skin evaluations.

### 2.5. Gastrointestinal System

Diarrhea, nausea, mucositis, stomatitis, dysgeusia, anorexia, abdominal discomfort, and weight loss may develop with the use of these drugs. Reduced side effects may occur if medication is taken with a large meal and water, if appropriate for administration per package insert. Appropriate use of supportive therapies with antidiarrheal or antiemetic medications may prevent the need for dose reduction or discontinuation. In the case of severe, unresponsive gastrointestinal effects, drug discontinuation should be implemented and reinitiated at a reduced dose once symptoms resolve to baseline or grade 1 level. Gastrointestinal perforation or fistula development is a rare, but potentially life-threatening, adverse event reported with TKIs. Risk factors include underlying tumor at perforation, diverticulitis, bowel obstruction, recent sigmoidoscopy or colonoscopy, and historical abdominal/pelvic irradiation [36]. Drug discontinuation is warranted if perforation event occurs. Consideration for a different TKI will need to be done with caution.

Hepatic toxicity or abnormalities, demonstrated by elevations in aspartate aminotransferase (AST) and alanine aminotransferase (ALT) and bilirubin, can occur. Elevations in AST or ALT were the most common metabolic abnormality requiring treatment seen in the phase III trial of pazopanib in renal cell carcinoma [28]. Although isolated elevations of total bilirubin were also seen at a similar frequency, concurrent elevations of ALT and total bilirubin were rare. The presence of a polymorphism in the uridine diphosphate glucuronosyltransferase 1A1 (UGT1A1) gene, which predisposes to Gilbert's syndrome, leads to reduced enzymatic activity necessary for the conjugation of bilirubin allowing it to be excreted in bile. Xu et al. reported that the presence of a polymorphism in UGT1A1 was significantly associated with pazopanib-induced hyperbilirubinemia, indicating that isolated unconjugated hyperbilirubinemia was a benign finding associated with Gilbert's syndrome, which did not require discontinuation of drug therapy [37]. Conjugated hyperbilirubinemia would require further investgation. None of the genetic markers evaluated in this study were associated with hepatic transaminase elevation, thus leaving the etiology still to be determined.

TKIs can lead to asymptomatic increases in pancreatic enzymes or rarely acute pancreatitis, most commonly reported with sorafenib and pazopanib. Standard treatment for pancreatitis and evaluation with endoscopic ultrasonography and other diagnostic testings for underlying causes of pancreatitis should be implemented. However, radiologic evidence of pancreatic damage or pancreatitis often is not found. Thus, dose-limiting toxicity for pancreatic enzyme elevation should be applied to grade 4 levels associated with clinical findings of pancreatitis, or if considered to be life threatening [38]. The cause of elevation in amylase and lipase is unclear, although some have attributed it to pancreatic ischemia from antiangiogenesis or to other drug-related effects.

### 2.6. Hematologic

Mucosal bleeding (e.g., epistaxis) to hemorrhage (i.e., gastrointestinal, pulmonary, cerebral, vaginal) has been reported with TKIs. Although mild mucosal bleeding could be attributed to inhibition of VEGFR-2 causing microvascular leaks from endothelial cell damage, clinically more severe hemorrhage is attributed to tumoral invasion of large vessels or other concurrent pathological conditions [36]. Additionally, thrombosis has been identified with TKI use. Inhibition of VEGF signaling could lead to overproduction of erythropoietin in the liver, which increases hematocrit and blood viscosity [39, 40]. Additionally, as wound healing is dependent upon angiogenesis, VEGF-inhibitors can impair or delay wound healing after surgery or other invasive procedures. Thus, drug should be withheld before and after surgery to optimize wound healing [36]. 

Hematologic laboratory abnormalities with neutropenia, lymphopenia, and thrombocytopenia are associated with TKIs. In contrast, anemia occurs less frequently, which may be explained by the relative increased erythrocytosis seen with this class of drugs. As patients with differentiated thyroid carcinoma may have received large cumulative doses of radioactive iodine and thyroid cancer patients may have received external beam radiation therapy, myelosuppression may be present prior to TKI initiation. Thus, routine monitoring of complete blood count and differential is required while on therapy.

### 2.7. Miscellaneous

Hypothyroidism or rising thyroid stimulating hormone (TSH), requiring increasing the thyroid hormone replacement doses, is seen as a class effect. Suggested etiologies have been poor absorption of levothyroxine from concomitant treatment-related diarrhea, or in patients with intact thyroid glands, regression of thyroid capillaries, or inhibition of thyroid peroxidase [36, 41]. Thyroid function should be monitored routinely while on TKI treatment to maintain a suppressed TSH in patients with DTC and a normal TSH in MTC patients.

Fatigue is a pervasive and often difficult-to-manage problem in cancer patients and may be related to many factors, in addition to direct toxicity of targeted drug therapy. Investigation for causes (e.g., anemia, hypothyroidism, cardiac dysfunction, renal dysfunction) should be performed. Supportive care with adequate nutrition, exercise, and stress reducing techniques is encouraged.

## 3. Recommendations for Dose Modifications or Discontinuation of TKIs due to Intolerance

**Nonhematologic Adverse Events (AEs)**

Patients with tolerable grade 1-2 nonhematologic AEs may continue TKI therapy while treatment for the AE is being optimized. For example, grade 1–3 hypertension does not necessarily require a dose modification or drug hold if the patient can be managed with antihypertensive agents. On the other hand, adverse events such as grade 1-2 skin rash, which have minimally effective treatments and/or are distressful or embarrassing to patients, may require drug interruptions. Although the package insert for sorafenib describes dose modification recommendation for cutaneous toxicity [8] (Table 3), others do not have clearly defined dose modifications for this toxicity. Recurrent grade 2 AEs require drug hold and often dose reduction if they are possibly related to the TKI and not responding to optimal supportive therapy. However, since TKIs are often chronic treatments for patients with thyroid cancer, the decision to hold and reduce the dose is often dictated in part by the patient's quality of life and physician judgment. Most grade 3 toxicities will require a drug hold until the AE improves significantly with resumption of the TKI at a reduced dose. However, grade 3 toxicities which can be readily managed (such as correction of hypokalemia arising from diarrhea which can be controlled) do not require a drug hold. Second occurrence of grade 3 toxicity should be managed again with drug hold and reduction of the dose. Third occurrences which cannot be effectively managed often require discontinuation of the TKI. Grade 4 AEs are life-threatening events, and if related to the TKI, require discontinuation of drug. However, in some select cases, it may be appropriate to resume treatment after reduction of the dose by two dose levels and if other interventions are implemented to prevent recurrence of the event. Thus, the decision to resume drug in patients with manageable grade 4 AEs, even when drug related, must be individualized and the benefit/risk ratio should be considered. Careful review of concomitant medications and herbal remedies which may cause increases in the drug levels of the TKI should also be given consideration.

**Hematologic Adverse Events**

Grade 2 hematologic toxicities do not require dose reduction. Grade 3-4 neutropenia and thrombocytopenia and grade 4 anemia require dose reductions upon first and second occurrences. Grade 3 and 4 hematologic toxicities are rare in thyroid cancer patients receiving TKIs; thus, other causes such as myelodysplasia should be ruled out.

**Intolerance to TKIs**

The definition of intolerance, proposed by Jabbour et al. in the context of leukemia, is met if the patient has one or more criterion as delineated in the manuscript [19]. We propose the following modified criteria as a definition of TKI intolerance: presence of one or more of the following criteria: (i) any grade 3-4 non-hematologic toxicity related to TKI therapy that has recurred despite dose reduction and optimal symptomatic measures, (ii) any grade 2 non-hematologic, intolerable toxicity, related to TKI therapy, that persists for more than a month despite optimal supportive measures, or (iii) grade 3-4 hematologic toxicity, related to TKI therapy, that is unresponsive to supportive measures and would require dose reductions below the accepted minimal effective dose, (iv) any life-threatening grade 4 non-hematological toxicity related to TKI therapy.

## 4. Variable Responses in Different Tissues

**Case Number 2**

**Figure 2:**
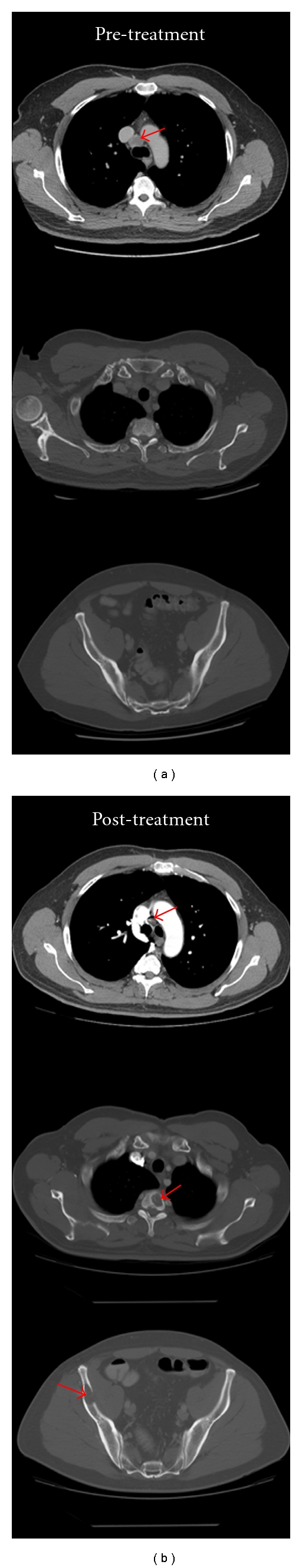
Patient with partial response in lymph nodes but progression in bone. CT scans before (a) and after (b) 6 months of therapy with a TKI. The patient had a partial response in mediastinal and hilar adenopathy but progression in bone with cortical destruction. The patient's bone lesions were irradiated, and he was restarted on the TKI. The patient continues on the TKI after 24 months and has no further evidence of progression.

A 54-year-old man with a history of stage IV papillary thyroid carcinoma was seen at our institution. He developed progressive disease that was noted to be nonavid to radioactive iodine. He was initiated on a clinical trial investigating a TKI in metastatic progressive thyroid carcinoma. He developed an excellent response (48% decrease in target lesion by RECIST), but his spinal bone metastasis continued to progress and became symptomatic (Figures 2(a) and 2(b)). His TKI therapy was held, and his progressive bone lesion was treated with external beam radiation. Due to overall favorable response in soft tissues, the TKI was restarted. The patient is still on therapy 24 months later with stabilization of disease in his bone and soft tissue lesions.

This case illustrates two points. First, tumor regression in response to TKI therapy can occur in some organs but not in other areas in the same patient. Additionally, TKI therapy can be continued in a patient with differential responses in various organs provided that local therapy is initiated for the region of progressive disease. This case is not unique; this scenario of varying responses to therapy in different organs is often encountered in metastatic thyroid cancer patients treated with TKI therapy. For example, lung metastases respond more favorably to sorafenib and sunitinib than do bone or pleura [42]. It has been noted that TKIs may lead to varying responses in different tissue sites in other cancers as well [43] and that continuation of systemic therapy after appropriate local therapy could be beneficial [44]. This differential response may not be unique to TKIs [45]. The pathophysiologic mechanism behind this variable response is not well elucidated. Some theories include host, tumor, and stroma factors. Resistance to TKI therapy has proven mechanisms in tumor and stroma as well. Some postulated theories include varying hepatocyte growth factor (HGF), VEGF receptors or serum levels, decreased drug bioavailability in certain organs, and organ-specific tumor resistance.

Until mechanisms are better elucidated to direct therapy for organ-specific TKI selection, consideration should be given to local therapies for areas of progressive disease. Clinically, one should consider irradiating bone lesions (especially if symptomatic) if they progress on TKI therapy. If a bone lesion is threatening vital structures (i.e., the spinal cord), consideration should be given to treating the bone lesion prior to TKI therapy. This may avoid a drug hold later and further compromise of vital structures. In general, the TKI is held during radiation therapy, although there are upcoming trials that may inform us differently. Bony metastatic lesion may also be treated with bisphosphonates or denosumab. This may decrease pain in the bony lesions or may decrease rate of progression, although trials are needed to determine efficacy of these therapies and frequency of dosing.

## 5. Sequential Use of TKIs

The former belief that if a patient has progressed through one TKI, he/she will fail with another TKI is false and outdated. Due to the many overlapping targets it was assumed that there would be complete cross-resistance. There is increasing evidence that with sequential application of these drugs, a patient who had progressive disease with one TKI may still respond to the next one. In a cohort of metastatic renal cell carcinoma treated with sunitinib after progression through sorafenib, the response rate (or efficacy) seen with second-line sunitinib after sorafenib was similar to that of first line sunitinib [46]. Investigations are under way to determine the best order for sequential TKI and other targeted therapies.

## 6. Summary

Drug development in oncology has led to several new targeted agents which have demonstrated efficacy in progressive thyroid cancer. Although it was initially thought that these drugs would prove to be less toxic than cytotoxic chemotherapy, the fact that these drugs have many off-target effects and the likelihood that most patients will be treated chronically beg the need for further research to better understand the cause of these toxicities and their optimal management. It also underscores the importance of appropriate patient selection. 

Patients and physicians must understand the possible adverse effects and weigh the advantages versus the risks of these drugs. Alternatives to systemic therapy for localized disease, such as external beam radiation or embolization should be considered when appropriate. Until prolongation of overall survival can be demonstrated with the use of the drugs, physicians should exercise caution in the selection of patients to undergo therapy with a TKI. 

Finally, more optimal drug selection should be personalized for the individual patient and tumor. Further research is needed to determine the ideal targeted therapy for an individual based on the molecular characterization of the tumor, stroma, and host factors. Future targeted therapy development may require that the on-target and off-target effects may be reengineered to enhance antiangiogenesis pathways and avoid cardiovascular, renal, and dermatologic pathways [47].

## Figures and Tables

**Table 1:** Major adverse events associated with commercially available TKIs which have been studied in thyroid cancer.

Adverse event	Sorafenib (%)		Sunitinib (%)		Pazopanib (%)		Vandetanib (%)	
	All-grade	≥grade 3	All-grade	≥grade 3	All-grade	≥grade 3	All-grade	≥grade 3
Hypertension	17	4	30	12	40	4	33	9
CHF or LVEF decline	1.7	NR	13	3	<1%	NR	<1	NR
Proteinuria	NR	NR	NR	NR	9	<1	10	0
Hand-foot skin reaction	30	6	29	6	6	NR	NR	NR
Stomatitis	NR	NR	30	1	4	NR	NR	NR
Anorexia	16	<1	34	2	22	2	21	4
Weight loss	10	<1	12	<1	52	3.5	10	1
Diarrhea	43	2	61	9	52	3.5	57	11
AST elevation	NR	NR	56	2	53	7.5	NR	NR
ALT elevation	NR	NR	51	2.5	53	12	51	2
Fatigue	37	5	54	11	19	2	24	6
Hypothyroidism	NR	NR	14	2	7	NR	NR	NR
Arterial thromboembolism	2.9	NR	NR	NR	3	2	NR	NR
Hemorrhage/bleeding (all sites)	15	3	30	3	13	2	NR	NR

CHF: congestive heart failure; LVEF: left ventricular ejection fraction; AST: aspartate aminotransferase; ALT: alanine aminotransferase; NR: not reported.

Data extracted from the phase 3 trials or from the prescribing drug reference information [9, 28–30].

Table is adapted from [31].

**Table 2:** Clinically significant CYP3A4 inducers, inhibitors, and substrates.

CYP3A4 inducers	CYP3A4 inhibitors	CYP3A4 substrates
Dexamethasone	Calcium channel blockers: amiodarone, verapamil	Statins: atorvastatin, lovastatin, and simvastatin (not pravastatin) (not rosuvastatin)

Anticonvulsants: phenytoin, carbamazepine	Azole antifungals: itraconazole, voriconazole, and ketoconazole	Calcium channel blockers: amlodipine, diltiazem, felodipine, nifedipine, and verapamil

Phenobarbital		

Rifampin	Macrolide antibiotics: erythromycin, and clarithromycin (not azithromycin)	

St. John's wort		

HIV antivirals: nonnucleoside reverse transcriptase inhibitors: efavirenz, and nevirapine	HIV antivirals: protease inhibitors: indinavir, nelfinavir, and ritonavir	

Pioglitazone		

**Table 3:** Suggested dose modification for skin toxicity for sorafenib [8].

Skin toxicity grade	Occurrence	Suggested dose modification
Grade 1: numbness, dysesthesia, paresthesia, tingling, painless swelling, erythema or discomfort of the hands or feet which does not disrupt the patient's normal activities	Any occurrence	Continue sorafenib and consider topical therapy for symptomatic relief

Grade 2: Painful erythema and swelling of the hands or feet and/or discomfort affecting the patient's normal activities	1st occurrence	Continue sorafenib and consider topical therapy for symptomatic relief. If no improvement within 7 days, see below
	No improvement within 7 days or 2nd or 3rd occurrence	Interrupt sorafenib until toxicity resolves to grade 0-1. When resuming treatment, decrease sorafenib dose by one dose level (400 mg daily or 400 mg every other day)
	4th occurrence	Discontinue sorafenib treatment

Grade 3: Moist desquamation, ulceration, blistering or severe pain of the hands or feet, or severe discomfort that causes the patient to be unable to work or perform activities of daily living	1st or 2nd occurrence	Interrupt sorafenib until toxicity resolves to grade 0-1. When resuming treatment, decrease sorafenib dose by one dose level (400 mg daily or 400 mg every other day)
	3rd occurrence	Discontinue sorafenib treatment
